# A twin with a novel pathogenic variant in *CYBB* induced X-linked chronic granulomatous disease: a rare case report of misdiagnosis as congenital cystic lung disease

**DOI:** 10.1186/s12887-026-06627-7

**Published:** 2026-02-21

**Authors:** Lin Lin, Zongrong Gong, Genquan Yin, Gen Lu, Junzheng Peng

**Affiliations:** Department of Respiration, Guangzhou Women and Children’s Medical Centre, Guangzhou Medical University, No.9, Jinsui Road, Zhujiang New City, Tianhe District, Guangzhou, Guangdong 510120 China

**Keywords:** Chronic granulomatous disease, Congenital cystic lung disease, *CYBB* gene

## Abstract

**Background:**

Chronic granulomatous disease (CGD) is a rare primary immunodeficiency disorder caused by defects in phagocyte NADPH oxidase. X-linked CGD, resulting from *CYBB* gene mutations, accounts for approximately two-thirds of cases. Clinical manifestations typically include recurrent bacterial and fungal infections, granuloma formation, and inflammatory complications.

**Case presentation:**

We report monochorionic diamniotic twins with a novel *CYBB* variant (c.1303A > T, p.Lys435Ter). The 7-month-old proband presented with recurrent respiratory infections and was initially misdiagnosed with congenital cystic lung disease. His twin brother carried the identical mutation but remained asymptomatic. Neutrophil function testing demonstrated impaired respiratory burst activity. Whole-exome sequencing confirmed a de novo nonsense mutation in the *CYBB* gene.

**Conclusions:**

This case highlights the diagnostic challenges of CGD and illustrates remarkable phenotypic discordance in monochorionic twins with identical genetic mutations. Environmental factors and epigenetic modifications may contribute to variable expressivity. Early genetic testing is crucial for accurate diagnosis and appropriate management of suspected primary immunodeficiency disorders.

## Background

Chronic granulomatous disease (CGD) was initially called “fatal granulomatous disease of childhood” in the 1950 s [1]. This primary immunodeficiency results from mutations in genes encoding NADPH oxidase complex components (*CYBB, CYBA, NCF1, NCF2*, and *NCF4*), impairing phagocytic reactive oxygen species production and intracellular pathogen killing [2]. Patients with CGD are susceptible to life-threatening infections with catalase-positive bacteria and fungi, such as *Staphylococcus aureus*, *Candida albicans*.

Congenital cystic lung disease represents a spectrum of rare developmental anomalies that may mimic CGD clinically through recurrent pulmonary infections [3, 4]. Herein, we report monochorionic diamniotic twins with X-linked CGD. The younger twin brother suffered from respiratory infection recurrently, who initially misdiagnosed with congenital cystic lung disease, whereas the older twin brother was asymptomatic.

## Case presentation

**Fig. 1:**
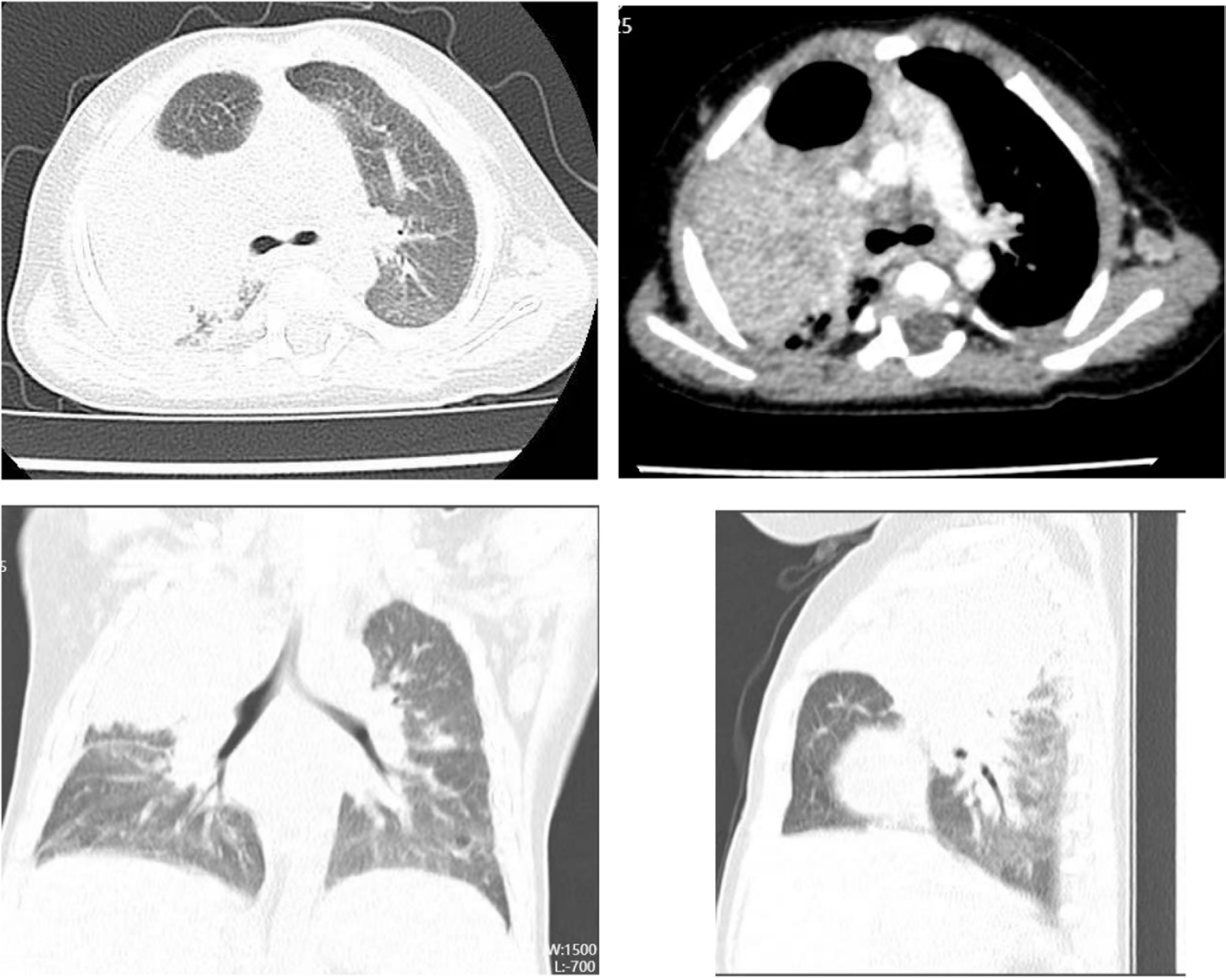
HRCT showing consolidation in the right upper lobe and multiple enlarged lymph nodes in the right hilum and mediastinum, as well as in the left axilla

**Fig. 2:**
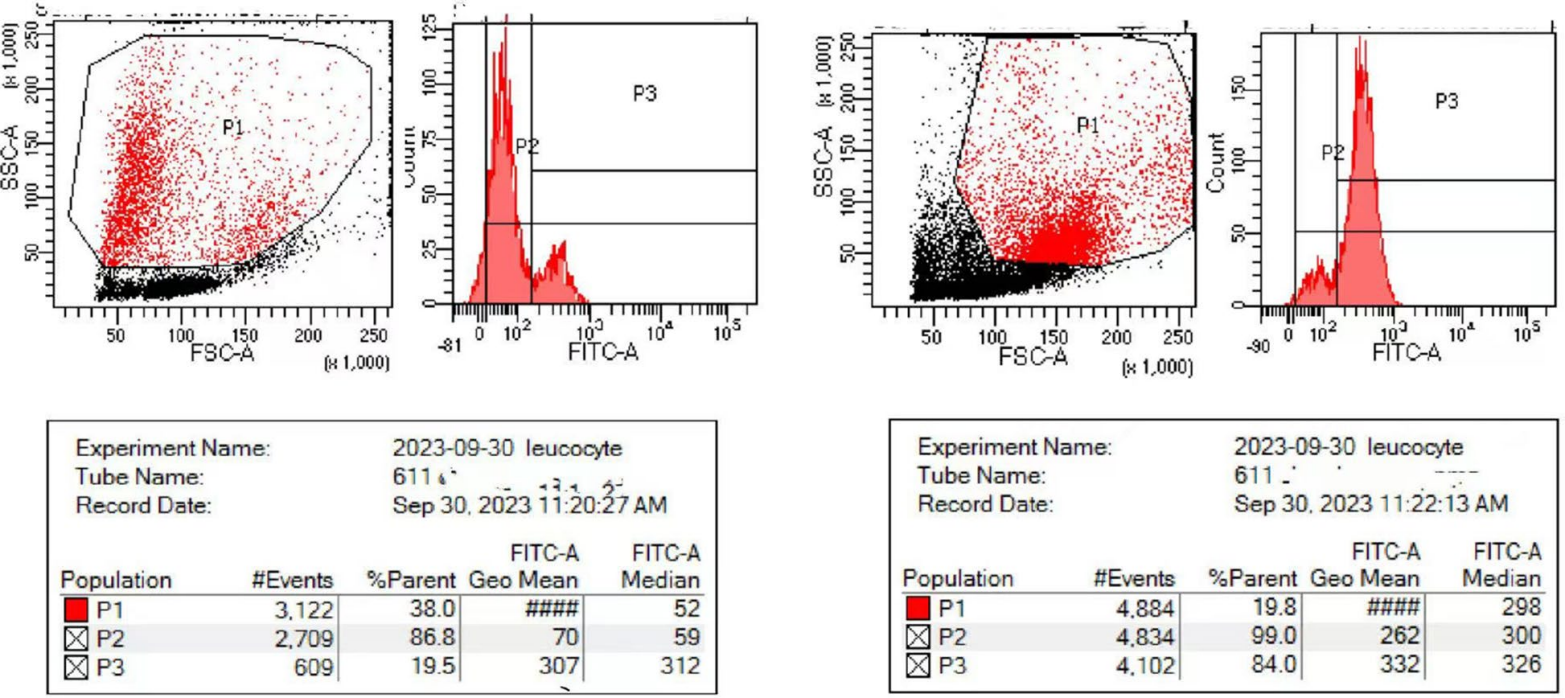
Neutrophil function after Phosphate Buffered Saline (PBS) and PMA stimulation (the peak DHR on the histogram unchanged after PMA stimulation)

In September 2023, a 7-month-old infant was admitted to the department of thoracic surgery for congenital cystic lung disease. The patient had experienced recurrent respiratory infections since early infancy. After admission, contrast-enhanced computed tomography (CECT) showed consolidation in the right upper lobe and multiple enlarged lymph nodes in the right hilum and mediastinum, as well as in the left axilla (Fig. 1). Notable history included the left upper limb was ulcerated and suppurated after Bacillus Calmette Guerin (BCG) vaccination. Immune deficiency could not be ruled out, therefore, he was transferred to the respiratory department for further treatment. He was the younger twin brother of monochorionic diamniotic twins, and his older twin brother who grew in the same environment, without history of recurrent pneumonia nor prophylactic treatment. There was no family history of primary immunodeficiency disease (PID) or consanguineous marriage. On admission, there was a slight old rash on the face, soybean-sized lymph nodes were palpated in the armpit, stridor and moist rales were revealed by auscultation in both lungs, and lymphadenopathy and hepatosplenomegaly were all absent. The patient’s neutrophil function test showed that the percentage and stimulation index was decreased after phorbol myristate acetate (PMA) stimulation, which means neutrophil activation was abnormal (Fig. 2,Table 1). Considering the patient’s previous history of recurrent pulmonary infections, bronchoscopy was performed with the consent of the parents, identified a small granuloma in the right upper lobe with little purulent secretion. Bronchoalveolar lavage fluid analysis detected human *parechovirus* (*HPeV*) but no *Staphylococcus aureus*, *Mycobacterium* or fungi. Ceftriaxone, linazolamide and aerosol inhalation of budesonide were administered from the first day of admission.

**Table 1:**
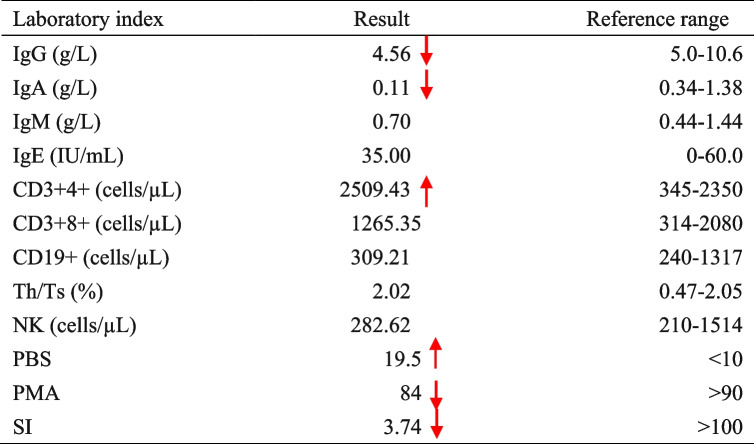
Laboratory FINDINGS of the patient on the day of admission

*WBC* white blood count, *N* neutrophils, *Hb* haemoglobin, *PLT* platelet count, *CRP* C-reactive protein, *Th* helper T cells. *Ts* inhibited T cells, *NK* natural killer, *PBS* phosphate buffered saline, *PMA* phorbol 12-myristate 13-acetate, *SI* stimulus index

After two weeks of treatment, the patient had no obvious fever or cough, but there was also no significant absorption of the lesion in the upper right lung. Further genetic testing from Beijing MyGenostics laboratory was performed, based on a decreased neutrophil respiratory burst and whole-exome sequencing revealed a de novo nonsense mutation, the hemizygous mutation of nucleotide 1303 from adenine A to thymine T (c.1303a > t) resulted in the change of amino acid 435 from lysine to terminator (p.lys435ter). His older twin brother had the same pathogenic variant, but his parents did not, it was a spontaneous pathogenic variant causing X-CGD. According to the variant interpretation guidelines developed by the American College of Medical Genetics and Genomics and the Association for Molecular Pathology, the mutation was considered pathogenic and had not been reported in the ClinVar database or dbSNP database and previous update [5].

## Follow-up

The diagnosis of X-CGD was evident, and the patient’s symptoms were currently stable. Therefore, he was discharged with linezolid and sulfamethoxazole. To date, the patient and his twin brother have been found to fully matched unrelated donors in the China Marrow Donor Program, and bone marrow transplantation will be performed. During the pre-transplant period, the patient received anti-tuberculosis treatment. Regular follow-up was maintained to monitor for infectious complications.

## Discussion

We present the case of a patient with X-linked CGD with a novel pathogenic variant in *CYBB* gene who was initially misdiagnosed with congenital cystic lung disease. His twin brother has the same pathogenic variant but was asymptomatic.

X-linked CGD caused by *CYBB* account for 65% to 70% of CGD cases in western countries, which encodes gp91phox. Our patient had a novel pathogenic variant in *CYBB*, and genetic analysis revealed that a hemizygous mutation of nucleotide 1303 from adenine A to thymine T (c.1303A > T) resulted in a change in amino acid 435 from lysine to terminator (p.Lys435Ter). His older twin brother had the same pathogenic variant, but his parents did not. Thus, it was a spontaneous pathogenic variant. This twin has almost identical genes, however, one twin suffers from certain diseases, and the other one asymptomatic. Our article is the first report about phenotypic variability in CGD. The same phenomenon occurs in cystic fibrosis [6], an autosomal recessive disease caused by a pathogenic variant in the *CFTR* gene, several factors may explain this phenotypic variability including: 1.environmental exposures: differential timing, dose, or type of infectious encounters—despite a shared household—may lead to varied immune activation and clinical outcomes [7, 8]. 2. epigenetic modifications: divergent DNA methylation, histone modifications, or non-coding RNA expression may alter the expression of immune-related genes, including *CYBB* or its regulatory elements, contributing to differential penetrance [9, 10]. 3.stochastic factors: random molecular or cellular events during hematopoiesis and immune cell differentiation could result in functional immune variation even in genetically identical individuals [11]. 4.microbiome differences: variations in the composition of the gut or respiratory microbiota—influenced by minor environmental or dietary differences—may modulate immune maturation and inflammatory responses, thereby affecting disease phenotype [12].

Dhydrorhodamine-1,2,3 assay could not detect carrier mother in de novo case with *CYBB* variant. Most X-CGD patients have the onset of symptoms before age 1 year [13]. The defective phagocytic NADPH oxidase-killing ability of pathogens results in repeated fungal and bacterial infections, leading to the formation of characteristic granulomas, a hallmark of the disease [14]. In our patient, electronic bronchoscopy examination shows a granuloma at the opening of the upper right lobe, which is prone to bleeding when touching. In addition to protective isolation, ceftriaxone and inazolamide were also utilized, creating time and opportunities for bone marrow transplantation for him. BCG vaccination is a contraindication in immunodeficiency, our patient had a history of routine BCG vaccination, and the axillary lymph nodes were enlarged without suppuration or ulceration. Chest CT indicated that the axillary lymph nodes of the pulmonary portal machine were enlarged. Although lymph node biopsy and acid-fast staining were not performed during this hospitalization, disseminated BCG disease was still considered [15]. His twin brother received BCG vaccination as well, as he was asymptomatic, and we did not know his lymph nodes enlarged or not. Before bone marrow transplantation, the patient received anti-tuberculosis treatment after being discharged from our hospital.

HR-CT of the patient from a local hospital revealed a mass density in the right upper lobe, so he initially sought treatment in the thoracic surgery department. However, contrast-enhanced CT at our hospital did not reveal the presence of cystic lesions, and in combination with the history of recurrent respiratory infections, the mass density was likely pulmonary consolidation. Congenital pulmonary airway malformation is not a common disease [16], and careful consideration before surgery is particularly important, especially for infants whose prenatal examination results are normal. Fortunately, the patient avoided surgery and received effective antibiotic treatment.

## Conclusion

In conclusion, we identified a novel pathogenic variant in an X-linked CGD infant, who was misdiagnosed as congenital cystic lung disease due to overlapping clinical features. His older twin brother had the same variant but asymptomatic, and environmental modifiers such as infectious microorganism that must be taken into account when dealing with this CGD case.

## Data Availability

All data and materials of this article are included in the manuscript and are thus available to the reader. The datasets generated and/or analysed during the current study are available in ：https://dataview.ncbi.nlm.nih.gov/object/PRJNA1174103?reviewer=k84es2qu3tm65smt20f8opihqb

## Abbreviations

BALF: Bronchoalveolar lavage fluid
BCG: Bacillus Calmette Guerin
CECT: Contrast-enhanced computed tomography
CGD: Chronic granulomatous disease
PBS: Phosphate Buffered Saline
PID: Primary immunodeficiency diseases
PMA: Phorbol Myristate Acetate
